# Genome-Wide Analyses for Osteosarcoma in Leonberger Dogs Reveal the *CDKN2A/B* Gene Locus as a Major Risk Locus

**DOI:** 10.3390/genes12121964

**Published:** 2021-12-09

**Authors:** Anna Letko, Katie M. Minor, Elaine M. Norton, Voichita D. Marinescu, Michaela Drögemüller, Emma Ivansson, Kate Megquier, Hyun Ji Noh, Mike Starkey, Steven G. Friedenberg, Kerstin Lindblad-Toh, James R. Mickelson, Cord Drögemüller

**Affiliations:** 1 Institute of Genetics, Vetsuisse Faculty, University of Bern, 3012 Bern, Switzerland; anna.letko@univ-rennes1.fr (A.L.); michaela.droegemueller@vetsuisse.unibe.ch (M.D.); 2 Institut de Génétique et Développement de Rennes (IGDR)—UMR6290, University Rennes, CNRS, 35000 Rennes, France; 3 Department of Veterinary and Biomedical Sciences, College of Veterinary Medicine, University of Minnesota, Saint Paul, MN 55108, USA; minork@umn.edu (K.M.M.); micke001@umn.edu (J.R.M.); 4 Animal and Comparative Biomedical Sciences, University of Arizona, Tucson, AZ 85721, USA; elainenorton@arizona.edu; 5 Science for Life Laboratory, Department of Medical Biochemistry and Microbiology, Uppsala University, 75105 Uppsala, Sweden; voichita.marinescu@imbim.uu.se (V.D.M.); emma.ivansson@igp.uu.se (E.I.); kmegq@broadinstitute.org (K.M.); kersli@broadinstitute.org (K.L.-T.); 6 Broad Institute of MIT and Harvard, Cambridge, MA 02142, USA; noh@broadinstitute.org; 7 Animal Health Trust, Newmarket CB8 7UU, UK; mike.starkey@aht.org.uk; 8 Department of Veterinary Clinical Sciences, College of Veterinary Medicine, University of Minnesota, Saint Paul, MN 55113, USA; fried255@umn.edu

**Keywords:** osteosarcoma, bone cancer, *canis familiaris*, Leonberger, animal model, *CDKN2A/B*

## Abstract

Dogs represent a unique spontaneous cancer model. Osteosarcoma (OSA) is the most common primary bone tumor in dogs (OMIA 001441-9615), and strongly resembles human forms of OSA. Several large- to giant-sized dog breeds, including the Leonberger, have a greatly increased risk of developing OSA. We performed genome-wide association analysis with high-density imputed SNP genotype data from 273 Leonberger cases with a median age of 8.1 [3.1–13.5] years and 365 controls older than eight years. This analysis revealed significant associations at the *CDKN2A/B* gene locus on canine chromosome 11, mirroring previous findings in other dog breeds, such as the greyhound, that also show an elevated risk for OSA. Heritability (h^2^_SNP_) was determined to be 20.6% (SE = 0.08; *p*-value = 5.7 × 10^−4^) based on a breed prevalence of 20%. The 2563 SNPs across the genome accounted for nearly all the h^2^_SNP_ of OSA, with 2183 SNPs of small effect, 316 SNPs of moderate effect, and 64 SNPs of large effect. As with many other cancers it is likely that regulatory, non-coding variants underlie the increased risk for cancer development. Our findings confirm a complex genetic basis of OSA, moderate heritability, and the crucial role of the *CDKN2A/B* locus leading to strong cancer predisposition in dogs. It will ultimately be interesting to study and compare the known genetic loci associated with canine OSA in human OSA.

## 1. Introduction

Osteosarcoma (OSA) is the most common primary form of bone cancer in children and adolescents but is a rare cancer overall [1,2]. Typically, OSA is located at the metaphysis of long bones. At the genomic level, structural variants are the driving mutagenesis events in pediatric osteosarcoma for which a complex karyotype is the hallmark [3]. Phenomena such as chromothripsis and kataegis, most often with somatic copy-number alterations in key oncogenes and tumor-suppressor genes have been found [2]. OSA predisposition in people is likely due to a combination of environmental factors, ethnicity, and genetic variants [4]. Rare familial sporadic forms of OSA have been reported to be caused by pathogenic variants in genes such as *CHEK2*, *RB1*, and *TP53* (OMIM 259500) with an essential function in cell survival pathways that play a role in genome stability (*RB1*, *TP53*, *CDK4*, *MDM2*, *ATRX*) [2]. However, the etiology of most human OSA is not yet known, the list of potential driving genes is growing, and it remains unclear whether genetic alterations in *TP53* or other genes are the cause or consequence of chromothripsis [2].

Osteosarcoma is the most common primary bone tumor in dogs [5] with incidence rates in some breeds 27 times higher than in people [6]. It is also clear that some breed histories and pedigree structures, coupled with increasing use of artificial selection, have resulted in many dog breeds with increased susceptibility to various forms of cancer [7]. Further, due to similarities in biology and treatment, dogs with OSA have been serving as an important model for the same disease in humans for many years, with veterinary and human medicine informing each other [8,9]. In contrast to human OSA, canine OSA appears to be heritable, e.g., in Scottish deerhound, the existence of a variant in a major gene with a dominant effect explaining most of the cases was postulated based on segregation analysis [10], similar to what was found recently in Irish wolfhounds [11]. While there is potential for any dog to develop OSA, some breeds appear to be more predisposed than others [5]. In a population of almost 400,000 insured Swedish dogs, 764 developed OSA and the top three breeds at highest risk were the Irish wolfhound, St. Bernard, and Leonberger [12]. Confirmed evidence of breed-related OSA risk and protection strongly supports studies aimed at defining the genetic basis for OSA initiation and pathogenesis [13]. However, the clade of the commonly OSA-affected large- to giant-sized breeds is large and contains other breeds that are not severely affected by OSA, so a universal or common origin for OSA seems less likely [14].

Several studies have sought to define the genetic bases for canine OSA susceptibility (OMIA 001441-9615). Initially, linkage analysis in a four-generation pedigree of Scottish deerhounds revealed a locus for OSA on dog chromosome 34 [15]. More recently, efforts with larger populations and whole-genome SNP genotype data have improved the understanding that certain breeds are predisposed to the development of OSA. In three breeds with a high incidence of OSA (Rottweiler, greyhound, and Irish wolfhound), a total of 33 inherited risk loci enriched for genes in pathways connected to bone differentiation and growth were found using GWAS, explaining 55% to 85% of phenotype variance in each breed [16]. Interestingly, the strongest association, located upstream of the *CDKN2A/B* genes on canine chromosome 11, is also the most rearranged locus in canine osteosarcoma tumors [16]. Subsequent DNA sequencing of tumors from dogs from three OSA-prone breeds (golden retriever, Rottweiler, and greyhound) revealed somatic mutation signatures similar to human OSA but also showing dog-specific differences [17]. This study highlights the strong genetic similarities between human and canine OSA could serve as an excellent model for developing treatment strategies for both species. Interestingly, the *CDKN2A* locus, among others, also influences the risk of developing histiocytic sarcoma in certain highly predisposed dog breeds [18,19].

This study aimed to extend previous studies by deciphering the genetic basis of OSA in Leonbergers. This breed has predispositions to hemangiosarcoma and osteosarcoma, among other forms of cancer, likely due in large part to limited genetic diversity [20]. Here we report a GWAS in a large population of Leonberger cases and controls using high-density imputed SNP genotype data. In addition, estimations of genomic architecture and heritability for OSA were performed. Our findings underline the crucial role of the *CDKN2A/B* locus on chromosome 11 leading to strong cancer predisposition in dogs.

## 2. Materials and Methods

### 2.1. Animals and Clinical Phenotypes

A total of 638 Leonbergers were included in this study, with 273 owner-reported OSA-affected cases and 365 owner-reported cancer-free controls eight years or older. These samples were collected by veterinarians during the medical care of the dogs with the informed consent of the owners. DNA was isolated from EDTA blood using standard methods.

### 2.2. SNP Array Genotyping and Genome-Wide Association Study

All samples were genotyped by using either the Axiom Canine Set A or HD arrays (Thermo Fisher Scientific, Waltham, MA, USA) or the Illumina CanineHD BeadChip array (Illumina, San Diego, CA, USA). Samples genotyped on lower density arrays were imputed with Beagle 4.1 [21] to 500 k density level as described previously [22]. Quality control filtering steps of the genotyping data were carried out with PLINK v1.9 [23]. The final dataset was pruned for low minor allele frequency (0.05) and failure to meet Hardy–Weinberg equilibrium (1 × 10^−6^) and consisted of 311,492 markers. A genome-wide association study (GWAS) was performed with GEMMA v0.98 [24], using a linear mixed model including an estimated kinship matrix from centered genotypes to account for population stratification and relatedness between dogs. The significance level of 0.05 was estimated by Bonferroni correction. All plots were generated in R environment v3.6.0 [25] with the qqman package [26]. All genome positions refer to the CanFam3.1 reference assembly. Fisher’s exact test was used as implemented in PLINK using the ‘—model fisher’ option to test for an association between a disease and a variant based on comparing allele frequencies between cases and controls.

### 2.3. Estimation of Heritability

SNP-based heritability (h^2^_SNP_) was estimated using the software program Genome-wide Complex Trait Analysis (GCTA) [27] from the imputed SNP markers after excluding the X chromosome. This program estimates narrow-sense heritability using a maximum likelihood statistic, which fits all SNPs at the same time. A genetic relationship matrix weighted for SNPs in linkage disequilibrium, generated from the software program Linkage Disequilbrium Adjusted Kinship [28], was included in the model to obtain an unbiased estimate of h^2^_SNP_. To correct for ascertainment bias inherent to case-control studies, estimates were transformed onto the liability scale using an estimated population prevalence of OSA at 20%. Although the true population prevalence of OSA is unknown, this value was extrapolated from our database of 1527 Leonbergers.

### 2.4. Determination of Genomic Architecture

In order to estimate the number of SNPs having a small, moderate or large effect, we used the software program BayesR [29] to determine the genomic architecture of OSA from the imputed SNP markers. This model estimates all SNPs simultaneously using Gibbs sampling under the assumption that the true SNP effect belongs to a mixture of four normal distributions of SNPs with no, small [10^−4^ × genetic variance (δ^2^_g_)], medium [10^−3^ × δ^2^_g_], or large [10^−2^ × δ^2^_g_] effect. The h^2^_SNP_ estimate generated above was included in the model to prevent inflation of the heritability estimate. Since BayesR estimates the total number of SNPs per bin based on the distribution of probabilities across all iterations, we utilized the modified code by Mollandin et al. [30] to extrapolate the per-SNP posterior variance.

## 3. Results

### 3.1. Animals

**Figure 1:**
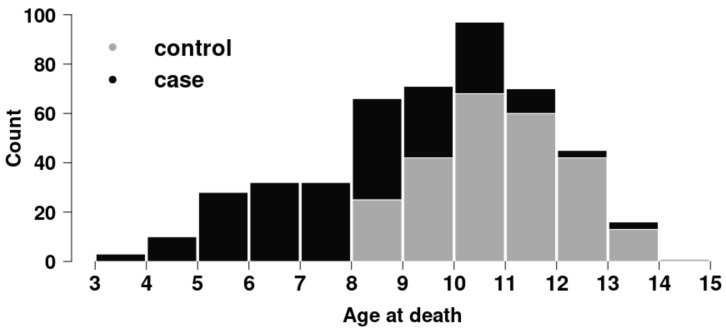
Distribution of age at death in 220 OSA-affected cases and 251 OSA-unaffected controls in Leonbergers.

Age at death information was available for a subset of 471 dogs (220 affected and 251 unaffected); the overall distribution is shown in Figure 1. The age at death in OSA cases ranged from 3.1 to 13.5 years (median of 8.1 years); and in controls, it ranged from 8.0 to 14.4 years (median of 10.9 years).

### 3.2. Genome-Wide Association Study

**Figure 2:**
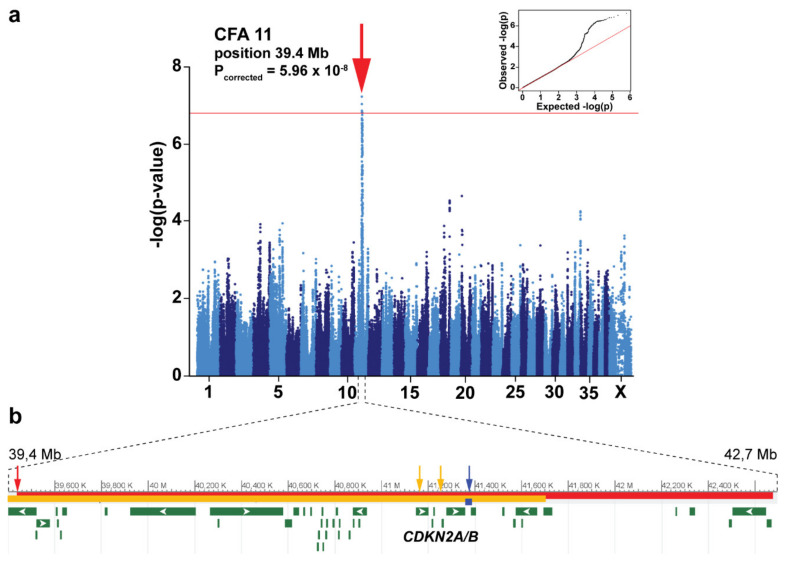
(**a**) Manhattan plot of the GWAS results using 273 OSA-cases and 365 controls shows an OSA-associated locus in Leonbergers on dog chromosome (CFA) 11. The −log *p*-values for each SNP are plotted on the y-axis and the position on each chromosome on the x-axis. The red line represents the Bonferroni-corrected significance threshold (−log(*p*-value) = 6.79). Inset: Corrected QQ plot confirms that the observed *p*-values of the best-associated markers have stronger association with the trait than expected by chance (null hypothesis, red line). (**b**) Genomic context of the associated locus on CFA 11 showing the association region and the lead SNP (red bar and arrow), the previously described region and lead SNP in OSA-affected greyhounds (blue bar and arrow) [16], the previously described region and lead SNPs in histiocytic sarcoma-affected Bernese mountain dogs, flat-coated retrievers and Rottweilers (orange bar and arrows) [19], as well as the candidate genes *CDKN2A/B*.

The GWAS with all 638 Leonbergers (273 affected dogs and 365 controls) revealed a single genome-wide significantly associated region for OSA on dog chromosome (CFA) 11 (Figure 2a; Table S1). The marker with the strongest association (*p*-value of 5.96 × 10^−8^) maps to CFA 11 position 39,434,964 (CanFam3.1). Additional suggestive association signals (*p*-values > 10^−4^) were visible on CFA 18, 20 and 33 (Figure 2a; Supplementary Table S1). The top ten best-associated markers (i.e., lead SNPs) defined a locus encompassing 3.2 Mb on CFA 11 (positions 39,434,964–42,676,919). This region contains 46 annotated genes and loci including the known cancer-associated cyclin-dependent kinase inhibitor encoding genes *CDKN2A* and *CDKN2B* (Figure 2b).

Subsequently, the age at death information was utilized to select a subset of younger cases (7 years or less, *n* = 77) and older controls (10 years or more, *n* = 184). GWAS performed using these cohorts revealed the strongest, but not genome-wide significant, association at the same CFA 11 locus as when using all animals, and further suggestive loci on CFA 4, 20, 22, 29, and 37 (Supplementary Figure S1a; Supplementary Table S1). The signal on CFA 20 does not overlap with the signal from the first analysis (full dataset GWAS: 5.8–7.9 Mb, subset GWAS: 48.5–49.5 Mb). The lead SNP position at CFA 11 was 42,238,647 (*p*-value of 1.22 × 10^−6^) in GWAS with all controls (*n* = 365) and younger cases (7 years or less, *n* = 77), and at 41,548,536 (*p*-value of 1.17 × 10^−5^) with older controls only (10 years or more, *n* = 184) compared to younger cases (Supplementary Figure S1b; Supplementary Table S1).

### 3.3. Allele Frequencies

Haplotype reconstruction over the CFA 11 locus revealed a large distribution of haplotypes among the entire population of cases and controls (Supplementary Table S2). To further define a risk haplotype, associated SNPs were filtered to produce a contiguous haplotype encompassing a region from chr 11:39,429,247–41,430,463, including the previously identified lead SNP in the greyhounds. The risk haplotype frequency among cases was 0.53 versus 0.38 in controls (chi-square *p*-value 9.02 × 10^−8^). Additionally, the frequencies of the lead SNPs were found to differ significantly between cases and controls (Table 1). The previously described lead SNP in OSA-affected greyhounds [16], as well as the previously described best-associated SNPs in histiocytic sarcoma-affected Bernese mountain dogs, flat-coated retrievers and Rottweilers [19] are shown in comparison (Table 1). The frequency of the lead SNP in greyhounds was previously investigated in eight more breeds with high rates of OSA including Leonbergers and showed the risk allele to be slightly more common in 30 cases (0.77) than 25 controls (0.62) [16]. Our data in a substantially larger population demonstrates similar frequencies of this SNP in cases and controls. Interestingly, the risk allele of the lead SNP for histiocytic sarcoma in Bernese mountain dogs was almost fixed in the investigated Leonberger groups (Table 1).

### 3.4. Heritability and Genetic Architecture

**Figure 3:**
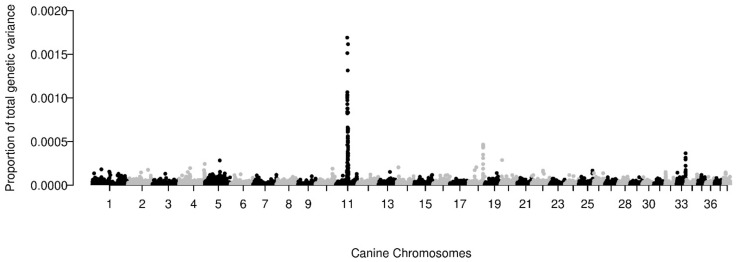
Posterior estimates of the total proportion of the estimated genetic variance explained by each SNP. The 38 canine autosomes are plotted on the x-axis and the estimated proportion of the total genetic variance explained is presented on the y-axis. Each dot represents a single SNP or overlapping SNPs.

Using a population prevalence of 20% for OSA, h^2^_SNP_ was estimated to be 20.6% (SE = 0.08; *p*-value = 5.7 × 10^−4^). The genomic architecture revealed that the h^2^_SNP_ was a contribution of 2563 SNPs with a small (*n* = 2183 SNPs), moderate (316 SNPs), and large effect size (64 SNPs). SNPs of moderate to large effect size accounted for 71% of the total genetic variance while SNPs of small effect size accounted for 29%. The modified BayesR code revealed that loci on CFA 11, 18, and 33 contributed the largest proportion of genetic variance. These regions were the same loci identified as significant (CFA 11) or suggestive (CFA 18 and 33) on GWAS. The loci on CFA 11, 18, and 33 accounted for 3.88%, 2.77%, and 1.06% of the total estimated proportion of genetic variance, respectively (Figure 3).

## 4. Discussion

Identification of inherited genetic risk factors is relatively easy in dogs due to low diversity and long linkage disequilibrium within breeds. Dogs and their owners also share environmental exposures that may be carcinogenic. Polygenic OSA risk may be high enough to appear Mendelian in certain dog breeds [31]. Nonetheless, there are several dog breeds that are known to have a genetic predisposition to OSA, but for which OSA-associated loci have yet to be identified [6]. Therefore, the aim of our project was to investigate the genetic variants responsible for the increased susceptibility of Leonbergers to OSA. Leonbergers have been found to be at increased risk of OSA in several geographic regions, including the USA and European countries [20]. We were able to collect 273 OSA-affected Leonbergers on both sides of the Atlantic Ocean over a period of 10 years that nicely recapitulate the predisposition for OSA in the general population. Therefore, we decided to study the Leonberger as an example of such breeds that could benefit from the identification of risk alleles for the development of heritable OSA.

Here, we present one of the largest GWAS of canine OSA using 365 carefully selected controls together representing the current diversity in Leonbergers, which confirmed the role of the *CDKN2A/B* locus as a major risk locus for OSA. Suggestive loci were also found on CFA 18, 20 and 33. Unfortunately, our data on a far larger cohort failed to confirm a higher allele frequency of the lead SNP on CFA 11 at position 41,375,800 (CanFam3.1) found in greyhounds and eight more breeds with high rates of OSA (including Leonbergers) [16]. However, our association signal spans 3.2 Mb, with the SNP with the strongest association located 1.6 Mb from the *CDKN2A/B* locus. While our signal is 1.8 Mb from the previously found signal, we hypothesize that both signals may be linked to the *CDKN2A/B* genes. Furthermore, our signal on CFA 11 was genome-wide significant, but we note that part of the signal comes from more related dogs than typically used for GWAS in humans. Our approach attempts to control for the relatedness, but it is worth remembering that this breed has a reduced rate of heterozygosity, which may contribute to the association through the inclusion of related dogs.

Cancer-predisposing loci may have pleiotropic effects and are associated with an increased risk of various cancers in various breeds. Here, we confirmed that the multi-cancer effect of the previously identified CFA 11 locus also influences the risk of OSA development in Leonbergers. In this regard, it was recently shown that *CDKN2A/B* was recurrently deleted in canine hemangiosarcoma tumors [32]. One might speculate that the recently reported age-related increase in the expression of *CDKN2A* in dogs diminishes a tumor-suppressive function that may account for the higher risk of developing OSA in certain aging dogs [33]. Among others, our results point toward these two strong candidate genes and to the possible existence of regulatory variants in the non-coding regions influencing the individual risk of developing OSA. Nonetheless, mapping of complex cancer in such a highly predisposed dog breed revealing further suggestive associations confirmed a polygenic spectrum of risk alleles indicative of specific pathways as drivers of canine OSA. It is hoped that these studies in dogs will help elucidate potentially targetable drivers. Nonetheless, their therapeutic benefits, both in animals and humans would need to be further addressed.

Our estimate of the genomic architecture revealed that nearly all of the genetic variance could be explained by 2563 SNPs, with the majority of these SNPs (85.2%) having a small effect and 14.8% (*n* = 380) having a moderate to large effect. Although fewer in number, the latter SNPs account for 71% of the genetic variation of OSA in our population. This distribution is consistent with that of a complex, polygenic disease [29]. This is further supported by our heritability estimate of 20.6%, indicating that additional environmental factors are having a significant impact on the phenotypic variation of OSA. It is also well recognized that large and giant breed dogs are at increased risk of OSA [34,35], as body mass has been strongly associated with OSA risk, although most reports of canine OSA are reported to occur in specific breeds [13]. This could point to the causal mutation events occurring before breed formation. Therefore, we suggest that future studies should take the height and weight information of OSA-affected dogs into account.

It is worth noting that our estimate of h^2^_SNP_ is likely underestimating the total genetic variation contributing to OSA. First, this is an estimate of narrow-sense heritability and only accounts for the additive effect of the genetic variants and does not factor in dominance or epistasis (i.e., broad-sense heritability). Second, SNP-based heritability estimates the genetic contribution of SNPs present on the genotyping array and does not account for other structural variants such as insertions, deletions, or copy number variants. Finally, due to a founder and popular sire effect, we have previously shown that Leonbergers have an average inbreeding coefficient of 0.28 indicating a dramatic loss in genetic diversity [20]. Thus, it is likely that many genetic variants contributing to OSA are fixed within the Leonberger population and would not be identified using mixed linear models, leading to an underestimate of h^2^_SNP_ as well as not being detectable on GWAS. Therefore, it would be valuable for future work to evaluate OSA risk alleles in the Leonberger population using FST or other selection-based statistics.

Importantly, the output of BayesR is not an estimate of the per-SNP effects, but the total number of SNPs which contribute to a phenotype based on a distribution of probabilities across iterations. Thus, we utilized a modification of the BayesR code to extrapolate the per-SNP posterior probability [30]. This allowed us to calculate the contribution of each SNP to the total h^2^_SNP_. The largest contributions to the total genetic variance were from loci on CFA 11, CFA 18 and CFA 33, all regions also found on our GWAS as associated with OSA. However, it is important to note that a true estimate of the proportion of heritability explained by a locus needs to be calculated from an independent population.

Across breeds of dogs, OSA is more likely to occur in middle-aged dogs, with an average age of onset of 7–10 years [36], and a smaller secondary peak occurring in young dogs (1–2 years) [35]. Thus, it is plausible that dogs with an earlier onset of OSA would have different risk alleles, or more variants with moderate to large effect, compared to dogs diagnosed at a later age. Leonbergers tend to follow this trend with an average age of onset of 7.8 years as well as a juvenile-onset [12]. We performed a GWAS with our Leonberger cases diagnosed at less than seven years of age, as well as restricting controls to over 10 years of age. When using only the younger cases, the top SNP on CFA 11 shifts to 42.2 Mb, and when, additionally, using only the oldest controls, the top SNP is at 41.5 Mb. No additional chromosomal loci became significant. This analysis does, however, have several limitations, including: (I) age of death is not an accurate representation of age of onset, (II) survival is influenced by stage at diagnosis, presence of metastatic disease, type and duration of treatment, (III) whether the owners opted to euthanize at the time of diagnosis [37], and (IV) this analysis greatly reduces the numbers of cases and controls, weakening our power to detect associations. The range of onset for middle-aged dogs is 7–10 years whereas juvenile onset is around two years of age, so our cutoff of less than seven years may have included both groups; we were underpowered to look specifically at the juvenile onset. Future studies designed to better address these two groups will increase our ability to identify genetic risk alleles for canine OSA.

In conclusion, OSA in Leonbergers is a moderately heritable complex disorder in which a major locus on CFA 11 contributes in combination with hundreds to thousands of other SNPs across the genome. Further work is needed to confirm the impact of the major genetic loci associated with OSA in Leonbergers and to explain the differences observed in the development of the fatal disorder. Furthermore, it will ultimately be interesting to study and compare the known genetic loci associated with canine OSA in human OSA.

## Figures and Tables

**Table 1:** Allele frequencies (AF) of OSA-associated SNPs on dog chromosome 11 identified in different studies in the current cohort of 273 cases and 365 controls.

Position	Alleles	Discovery Breed	AF in Leonbergers		Leonberger *p*-Value ^1^	Reference
			Cases	Controls		
39,434,964	G/A	Leonberger	0.64	0.47	1.64 × 10^−9^	current study
41,375,800	C/A	Greyhound	0.73	0.63	1.16 × 10^−4^	[16]
41,161,441	T/C	Bernese mountain dog	0.93	0.95	0.16	[19]
41,252,822	T/C	Flat-coated retriever, Rottweiler	0.71	0.61	1.15 × 10^−4^	[19]

^1^ Fisher’s exact test.

## Data Availability

The SNP data analyzed during the current study are not publicly available but are available from the corresponding author on reasonable request.

## Supplementary Materials

The following are available online at https://www.mdpi.com/article/10.3390/genes12121964/s1, Table S1: The 10,000 most significant markers sorted by *p*-value obtained from the genome-wide association studies. Table S2: Phased haplotypes for 1147 SNPs in 638 Leonbergers. Figure S1: Manhattan plots of the GWAS results for OSA using either (**a**) all controls (*n* = 365) and younger cases (7 years or less, *n* = 77) or (**b**) older controls (10 years or more, *n* = 184) and younger cases (7 years or less, *n* = 77).
