# Supplementary material for: Genome-Wide Analyses for Osteosarcoma in Leonberger Dogs Reveal the CDKN2A/B Gene Locus as a Major Risk Locus
Source: Genes (Basel). 2021 Dec 9;12(12):1964. doi: 10.3390/genes12121964 (PMC8700858; doi:10.3390/genes12121964)
Supplement: Supplementary file 1 [file genes-12-01964-s001.zip › Figure S1.pdf]

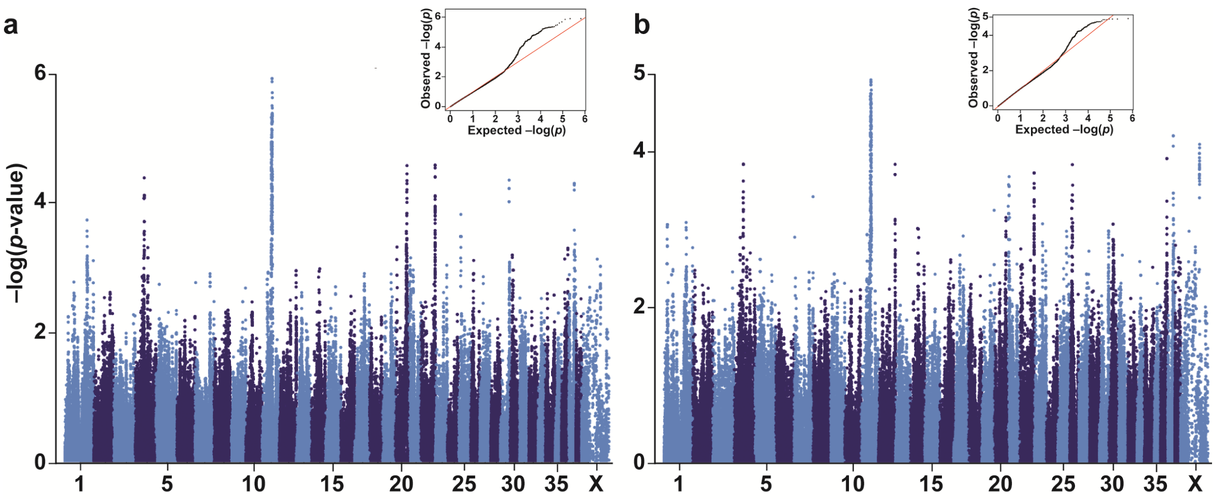

Figure S1: Manhattan plots of the GWAS results for OSA using either (a) all controls (*n* = 365) and younger cases (7 years or less, *n* = 77) or (b) older controls (10 years or more, *n* = 184) and younger cases (7 years or less, *n* = 77).
